# Necrotizing Fasciitis Complicating Pregnancy: A Case Report and Literature Review

**DOI:** 10.1155/2014/505410

**Published:** 2014-03-09

**Authors:** Marinos Nikolaou, Petros Zampakis, Vasiliki Vervita, Konstantinos Almaloglou, Georgios Adonakis, Markos Marangos, Georgios Decavalas

**Affiliations:** ^1^Department of Obstetrics and Gynecology, Medical School, University of Patras, Rio Achaia, 26500 Patras, Greece; ^2^Department of Radiology, Medical School, University of Patras, 26500 Patras, Greece; ^3^Department of Internal Medicine, Division of Infectious Diseases, Medical School, University of Patras, 26500 Patras, Greece

## Abstract

Necrotizing fasciitis is a rare, life-threatening surgical infection in pregnancy with high rates of morbidity and mortality. A 15-year-old primigravid woman, at 28 weeks of gestation with no significant previous medical history, was admitted to our hospital complaining of severe left lower extremity pain and high fever the last 72 hours. During clinical examination, she had a swollen, erythematous and tender to palpation inflamed skin over the medial aspect of the upper thigh without any evidence of injury. Incision drainage was performed immediately and she received broad spectrum antibiotics. During initial laboratory examinations, diabetes mellitus was diagnosed. There was no clinical improvement over the following days. Magnetic resonance imaging (MRI) revealed subcutaneous tissue inflammation and edema of infected tissues confirming the disease entity. Multidisciplinary therapy with immediate aggressive surgical debridement of necrotic tissues, multiple antibiotics, and intensive care monitoring was performed successfully. The patient's postoperative course was uncomplicated and skin defect was closed with split thickness skin grafting. Our case emphasized the potential immunosuppressive role of pregnancy state in conjunction with diabetes mellitus in the development of severe necrotizing soft tissue infections.

## 1. Introduction

Necrotizing fasciitis (NF) is a rare life-threatening invasive soft tissue infection which is characterized by widespread necrosis of subcutaneous tissue, superficial fascia, and other adjacent tissue [1]. It is a surgical emergency with reported overall high mortality rate among patients with NF up to 76% [2]. It primarily involves the subcutaneous tissue and rapidly extends along superficial fascia planes [3]. Management of NF is based on early, aggressive surgical debridement of necrotic tissues, broad spectrum antibiotics, and intensive supportive care [3, 4].

Numerous aerobic and anaerobic pathogens are synergistically implicated in the pathogenesis of disease [1, 5]. NF occurs mainly in patients with predisposing factors such as diabetes mellitus, obesity, peripheral vascular disease, and immune system impairment or following a variety of injuries and surgical procedures which result in skin integrity interruption and rarely from hematogenous spread [2, 5].

Pregnancy is responsible for an immunosuppressive state, which may contribute to the development of severe necrotic soft tissue infections [6, 7]. Previous studies have showed that NF in pregnancy is rare and usually is characterized by acute onset and rapid clinical progression involving the vulva, perineum, lower extremities, and abdominal wall of the pregnant or postpartum women [2, 8].

We report a rare case of rapidly progressive NF complicating a young pregnant woman.

## 2. Case Report

A 15-year-old primigravid woman, at the 28th week of gestation, presented with a 3-day history of severe left thigh pain and high fever with chills to the emergency department. Physical examination revealed clinical signs of severe soft tissue infection with erythema, edema, and extreme tenderness of the skin over the medial aspect of the left upper thigh. The vital signs were temperature of 39.5°C; pulse rate 115 beats/min; blood pressure 90/60 mmHg; respiratory rate 30/min. The laboratory results on admission were as follows: white blood cell (WBC) count of 24,000/*μ*L with 84 band forms, serum sodium 126 mmol/L, blood urea nitrogen (BUN) level of 23 mg/dL, C-reactive protein (CRP) of 29.5 mg/dL, and blood fasting glucose of 369 mg/dL.

She denied any recent trauma and her past medical history was unremarkable. She complained of an increase thirst and urination. Ultrasound obstetric exanimation performed at admission revealed a living, intrauterine fetus of 28 weeks gestational age. A venous Doppler examination of the lower extremities was normal without signs of deep venous thrombosis (DVT).

A skin abscess formation was suspected and she was initially treated with incision and drainage of pus exudates. She was started on empiric IV antibiotic coverage (amoxicillin-clavulanic acid 1.5 grams every 8 hours), IV fluids for correction of electrolytes, and rapid and long-action insulin for diabetes mellitus.

**Figure 1:**
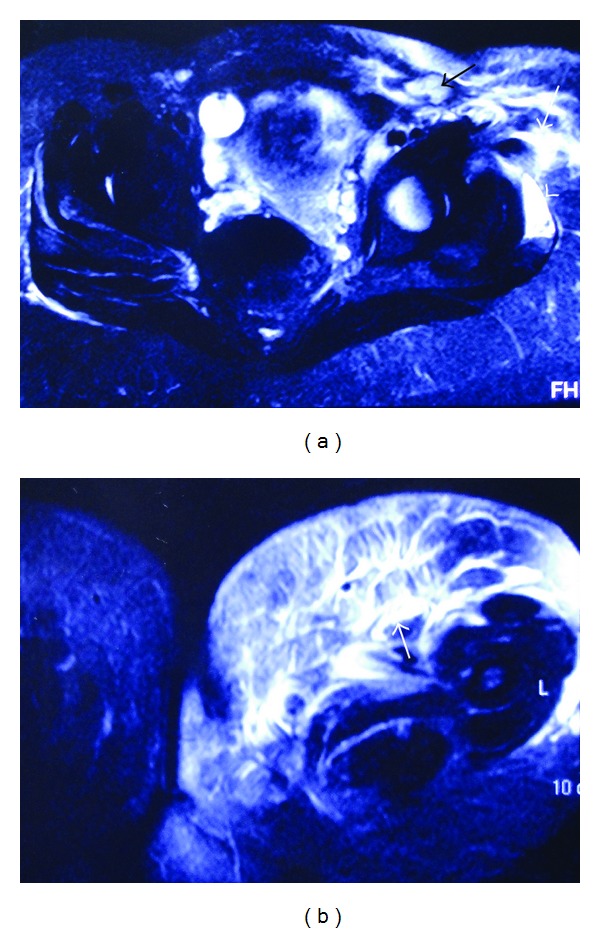
((a)-(b)) Axial T2W images at the level of acetabulum (a) and upper thigh (b). White arrows show high signal areas at the soft tissues of the anteromedial part of left thigh, indicating inflammatory process. White arrowhead shows high signal within the fascia next to the left gluteus maximus muscle. Inflammatory lymph node is seen at the left groin (black arrow).

**Figure 2:**
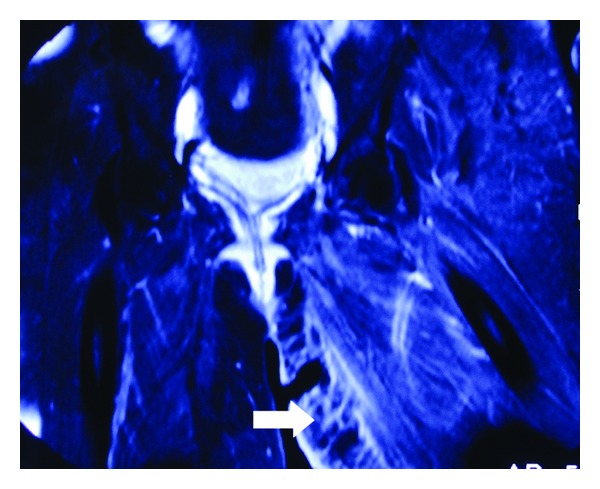
Coronal T1W SPIR images, following Gadolinium administration, show enhancement of the medial part of the left thigh, next to the previously performed surgical wound (white thick arrow).

**Figure 3:**
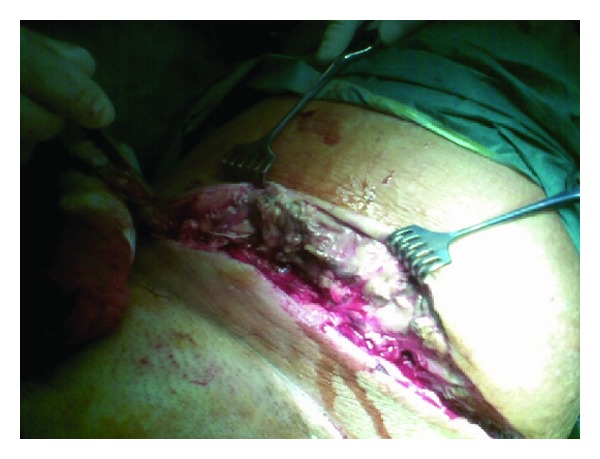
Extensive surgical incision required for initial debridement of necrotizing soft tissue infection.

However, the patient had no clinical and laboratory improvement in the following 24 hours. Her temperature, WBC and count and CRP increased to 39.9 C, 27,000 *μ*/L and 31.8 mg/dL, respectively. Wound cultures grew *Escherichia coli *and* Staphylococcus epidermidis*. The patient was administered Vancomycin (1 gram every 12 hours) and Meropenem (1 gram every 8 hours). On the third hospital day, the patient was scanned at the MRI unit of the Department of Radiology, University Hospital of Patras, by means of a Philips Medical Systems 1T MRI scanner. Axial and coronal images were obtained. High signal areas at the soft tissues of the anteromedial part of left thigh, indicating inflammatory process, were visualized (Figures 1(a)-1(b) and 2). An emergency radical surgical debridement of infected necrotic tissue was performed involving skin, subcutaneous tissue, and fascia of the anterior-medial compartment of the anterior-medial compartment of the thigh up to the inguinal area (Figure 3). The wound was packed open with gauze moistened with saline. She required four additional intraoperative debridement on daily basis until progression of disease had been halted and all necrotic tissue had been removed. On the fifth postoperative day, her WBC increased up to 31,000/*μ*L with high fever of 39.5 C and an altered level of consciousness was noted. She was transferred to the intensive care unit for close monitoring and support of vital functions. She started to have preterm uterine contractions despite tocolytic therapy and delivered a viable male fetus weighing 1470 grams by normal labor. The baby died to neonatal intensive care unit due to septicaemia after 48 hours.

Intraoperative culture of infected tissue grew *Enterococcus faecalis, Acinetobacter, *and* Candida albicans.* Based on the sensitivity of the microorganisms from tissue cultures, we administered meropenem 2 gr × 3 daily, linezolid 600 mg × 2 daily, metronidazole 500 mg × 3 daily, and liposomal amphotericin B 300 mg once a day IV.

Finally, thirty-eight days after the initial debridement, the patient continued to improve clinically, and she was transferred to a plastic centre facility for reconstruction of the wound. The wound was covered with split-thickness skin graft. Ten days later, the patient was discharged and currently is well. The source of infection remains unclear.

## 3. Discussion

NF is a life-threatening surgical emergency. It is a severe, potentially fatal infectious disease which rapidly extends from the subcutaneous tissue along the superficial and deep fascia causing vascular occlusion, ischemia, and necrosis of tissues [1–5]. Bacterial endotoxins with the release of cytokines are mediators of rapid tissue destruction and have a crucial role in progression of the disease [1, 4, 5].

Predisposing factors for the development of NF are well documented [1–5]. Diabetes mellitus and pregnancy represent two main poor prognostic factors. In the analysis of series patients with NF who were admitted to gynecology and obstetrics services, diabetes mellitus was noted in 34.7% of cases [2]. In our case, diabetes mellitus was diagnosed incidentally at the time of hospital admission. Pregnancy itself represents an additional risk factor due to suppression of immune system during the second and third trimester and in postpartum period [6, 7].

The clinical presentation of NF is often characteristic, including high fever with chills, signs of systemic toxicity, and severe pain. Without prompt and urgent therapeutic intervention, it may rapidly lead to septic shock syndrome with cyanosis, hypotension and tachycardia, altered level of consciousness, multiorgan failure, and death [4, 5]. The inflamed skin appears erythematic with edema and blistering but its involvement is smaller than the extent of necrosis of the underlying subcutaneous tissue and fascia, making the clinical distinction between simple cellulitis and NF extremely difficult [1, 4, 5, 9]. On admission, our patient had local inflammatory skin changes, clinical signs of sepsis and high fever.

The definitive diagnosis of NF is made after surgical debridement with microbiologic and histological examination of infected tissues [4, 5, 9, 10].

New diagnostic methods of NF have been pursued with modern imaging modalities [1–3, 5]. The imaging findings preceded the development of cutaneous signs of underlying infection offering an important diagnostic adjunct to the urgent management of this life-threatening condition [11, 12]. MRI appears specific to determine with diagnostic accuracy the extent of fascia inflammation, the impending infective tissue necrosis, and the need for urgent surgical management in patients with NF in the lower extremity [13]. Early diagnosis of NF in our patient was based on clinical suspicion followed by MRI which permitted visualization of subcutaneous tissue edema and inflammatory process in the anterior medial aspect of thigh.

Prognosis of patients with NF depends on early diagnosis and aggressive multidisciplinary management based on deep surgical debridement of all necrotic tissues, intravenous antibiotics, fluids and electrolytes management, appropriate analgesia, and intensive care support [1–5, 9, 10, 14]. Daily evaluation of the open wound with irrigation and aggressive surgical debridement until infection is halted was notably associated with reduced mortality [3, 5, 9, 15, 16]. Mortality rate varies widely in the series of patients with NF ranges from 6% to 76% [2–5, 9]. Among risk factors of mortality in patients with NF, a delay in recognition and inadequate operative debridement had a significant negative impact on outcome and was associated with increased morbidity and mortality [5, 10].

Broad-spectrum intravenous antibiotics should be empirically administrated before the results of cultures to cover gram-positive cocci, gram-negative enteric rods, and anaerobic flora [1–5, 9, 10]. They can chance according to culture results and clinical response of the patient [5, 9, 10]. In series of patients with NF, a predominance of synergistic polymicrobial infections was cultured from affected wounds [2, 5, 9, 10].

Recently, hyperbaric oxygen (HBO) therapy is used as an adjunct therapy in NF to improve tissue oxygenation necessary for normal control healing. However, its efficacy remains unclear due to lack of well controlled, randomized, clinical trials [2, 5, 10, 17]. Furthermore, the administration of intravenous immunoglobulin (IVIG) was found by several researchers to be clinically beneficial in terms of survivalin patients with NF [5, 18].

On conclusion, our case emphasized the potential immunosuppressive role of pregnancy state in conjunction with diabetes mellitus to development of severe necrotizing soft tissue infections. Early recognition, determination of the extent of necrosis, appropriate aggressive therapy, and intensive care support are the most important predictors of survival in patients with NF.
